# In-Vehicle Time-Sensitive Networking with Blockchain-Based Error-Bounded Data Management

**DOI:** 10.3390/s26134260

**Published:** 2026-07-04

**Authors:** Ray-I Chang, Ting-Wei Hsu, Yu-Han Ke

**Affiliations:** Department of Engineering Science and Ocean Engineering, National Taiwan University, Taipei 10617, Taiwan; f11525125@ntu.edu.tw (T.-W.H.); r14921091@ntu.edu.tw (Y.-H.K.)

**Keywords:** IoT, LiDAR, data engineering, blockchain, IPFS, verifiable archival, Time-Sensitive Networking, autonomous driving systems, in-vehicle networks, distributed storage

## Abstract

Autonomous driving systems (ADSs) increasingly rely on LiDAR sensors for perception. However, the resulting high-volume data places a strain on storage systems and network bandwidth and raises data-privacy concerns. We propose an IoT data engineering framework for processing, transmitting, storing, and retrieving high-volume LiDAR sensor data in in-vehicle systems that combines error-bounded compression and blockchain-based storage over in-vehicle Time-Sensitive Networking (TSN). With IEEE 802.1Qbv-based TSN scheduling, our framework supports deterministic delivery within the evaluated setup. It combines AES-GCM encryption, blockchain smart contracts, and InterPlanetary File System (IPFS) storage to support confidential, tamper-evident archival under the stated trust and threat model. Experimental evaluation on the KITTI dataset demonstrates that our BEDM framework reduces LiDAR data volume by 75.4%, contributing to a total network bandwidth reduction of 53.7%. The results demonstrate the feasibility and effectiveness of the integrated framework within the evaluated KITTI-based setup and single-switch TSN abstraction, and cross-scene and TSN traffic-sensitivity analyses further characterize its robustness.

## 1. Introduction

Autonomous driving systems (ADSs) equipped with modern sensor suites face demanding data management requirements. A single vehicle with multiple LiDAR sensors can generate hundreds of megabits per second of point cloud data, with the full sensor suite producing on the order of tens of terabytes per day, based on industry and prior estimates [1,2], consuming significant portions of available network bandwidth. This creates three bottlenecks: (i) network bandwidth saturation that prevents the integration of additional sensors, (ii) prohibitive storage costs that challenge economic viability, and (iii) data privacy concerns in increasingly connected vehicle ecosystems [3,4]. These problems limit ADS scalability and call for a solution that jointly addresses data volume, transmission determinism, and security. From an IoT perspective, the bottleneck in these sensor-rich platforms has shifted from sensing to the engineering of high-volume data for timely transmission, secure storage, and reliable retrieval.

Current approaches address these challenges in isolation. Point cloud compression methods such as Draco [5] and Point Cloud Library (PCL) compression [6] achieve high compression ratios; however, they lack formal error-bound guarantees, as users select quantization or quality parameters rather than certified bounds. Our previous work demonstrated error-bounded LiDAR compression, which achieved 25–35% of the original size [7], but it was not integrated with network or storage infrastructure. Time-Sensitive Networking (TSN) provides deterministic communication [8,9], but existing deployments typically assume static bandwidth requirements with fixed periods and frame lengths [10,11], and cannot adapt to variable compression ratios. Blockchain-based vehicle data management systems [12,13] ensure data integrity but struggle with the volume of sensor data. Without integration, these technologies leave the underlying problem largely unresolved.

TSN, developed by the IEEE 802.1 Task Group, enables deterministic Ethernet communication essential for ADSs [14]. IEEE 802.1AS provides sub-microsecond time synchronization through the Precision Time Protocol (PTP) [8,9], while IEEE 802.1Qbv defines Time-Aware Shaper (TAS) mechanisms for deterministic scheduling [8,9]. Together, they provide guaranteed bandwidth allocation and bounded latency. However, as shown in classic TSN scheduling models [10,11], existing approaches assume fixed stream parameters, limiting their adaptability to dynamic compression scenarios. Simulation studies confirm these limitations in automotive contexts [15].

Blockchain technology and the InterPlanetary File System (IPFS) provide decentralized data integrity and storage [16]. Smart contracts automate data verification and access control, while IPFS provides content-addressed distributed storage that scales beyond traditional architectures [16]. Recent work on vehicular networks shows that blockchain can support reliable data storage using IPFS [12,13] and trust management [17], but applying these technologies to high-bandwidth sensor data is still largely unexplored.

We propose a blockchain-based error-bounded data management (BEDM) framework that combines error-bounded compression and blockchain-based storage over in-vehicle TSN to address the data management challenges of ADSs. With IEEE 802.1Qbv-based TSN scheduling, the framework supports deterministic delivery within the evaluated setup. It also uses blockchain smart contracts with IPFS distributed storage to support data confidentiality and tamper-evident archival. Evaluation on the KITTI dataset [18] indicates the feasibility of the integrated BEDM pipeline within the evaluated setup, while leaving broader generalization to additional datasets, topologies, and traffic patterns for future work. The main contributions of this paper are as follows: (1) we present a system-level co-design that integrates error-bounded LiDAR compression, TSN-based deterministic delivery, and blockchain/IPFS-based verifiable archival into a single data pipeline; (2) we characterize the cross-layer interaction between bounded-error compression and TSN scheduling, quantifying how compression changes the in-vehicle stream set and link utilization presented to the TSN scheduler; (3) we design a blockchain/IPFS-based verifiable archival path that stores encrypted compressed LiDAR payloads off-chain while anchoring compact verification metadata on-chain through asynchronous, Merkle-batched commitments, thereby preserving auditability without placing blockchain confirmation on the real-time control path; and (4) within the current experimental setup, we provide a cross-layer evaluation covering bandwidth, scheduling, and storage, including supplementary cross-scene and limited TSN traffic-sensitivity analyses to probe robustness.

This work is organized around three research questions, each with an explicit evaluation criterion. RQ1—can bounded-error LiDAR compression reduce in-vehicle network and storage load while keeping safety-critical streams schedulable under IEEE 802.1Qbv? (criteria: bandwidth and data-volume reduction, link utilization, and TSN schedulability). RQ2—does bounded-error compression preserve reconstruction fidelity within a declared, certifiable bound? (criteria: maximum per-point error relative to the declared bound, Occupancy IoU, and Chamfer distance). RQ3—can verifiable, tamper-evident archival be provided off the real-time control path at a reproducible and bounded cost? (criteria: on-chain gas, archival and verification latency, and the pass rate of executable negative tests). The associated hypothesis—that the integrated pipeline simultaneously satisfies these bandwidth, fidelity, and integrity criteria within the evaluated setup—is tested in Section 4.

The blockchain and IPFS components are not proposed as a new distributed ledger or storage protocol; both are mature off-the-shelf technologies, and their integration has been explored in numerous prior works. The contribution of this work lies in the cross-layer integration of error-bounded LiDAR compression, deterministic TSN delivery, and a blockchain/IPFS-based verifiable archival layer in a single data pipeline, with a quantified, threat-specific evaluation of each component. BEDM uses blockchain as an asynchronous tamper-evident anchoring layer rather than as a real-time control-path component.

Section 2 reviews related work. Section 3 presents the system architecture. Section 4 describes the implementation and evaluation. Section 5 discusses the results. Section 6 concludes this paper.

## 2. Related Works

### 2.1. LiDAR Point Cloud Compression

The rapid growth of LiDAR data in ADSs has motivated research into point cloud compression. Traditional lossless compression methods such as LASzip preserve all geometric information and were designed for archival purposes, with LASzip additionally supporting streaming access [19,20]. While these methods guarantee perfect reconstruction, they cannot meet the bandwidth and latency requirements of ADSs, where multiple LiDAR sensors generate hundreds of megabits per second [1,2].

General-purpose lossy compression offers more aggressive data reduction. Google’s Draco [5] employs quantization and entropy coding, while PCL [6] provides octree-based compression with configurable precision. The MPEG Geometry-based Point Cloud Compression (G-PCC) standard offers both lossless and lossy modes optimized for point cloud geometry and attributes [21,22]. However, these methods do not provide formal error-bound guarantees; users select quantization or quality parameters, and the effect on perception algorithms is hard to predict.

Error-bounded compression, originally developed for scientific computing, provides mathematically guaranteed error limits. For example, SZ combines best-fit curve fitting with entropy coding under user-specified error bounds to achieve high compression ratios on high-performance-computing floating-point arrays [23], while ZFP adopts a fixed-rate block-based representation that supports efficient random access for a prescribed per-block bit budget [24]. Together, they demonstrate that controlling maximum point-wise error enables predictable quality degradation while achieving significant data reduction.

In our previous work [7], we extended this concept specifically to LiDAR point clouds, introducing the EB-HC (Error-Bounded Huffman Coding), EB-3D (Error-Bounded 3D compression), and EB-HC-3D (EB-HC with 3D integration) algorithms. These methods compress to 25–35% of the original size for single-frame scenarios and 15–25% for multi-frame scenarios while maintaining a strict 2 cm error bound. However, that work addressed only the compression algorithms, without considering deterministic network transmission or secure storage.

Recent advances in learning-based compression, such as OctSqueeze [25] and RIDDLE [26], show promise with competitive compression ratios but can incur substantial computational cost that may be difficult to reconcile with real-time automotive constraints. The trade-off between compression ratio, computational cost, and error guarantees remains open, especially for in-vehicle edge devices under deterministic networking. In this work, we adopt error-bounded compression specifically for its certified per-point guarantee rather than for maximal ratio: general-purpose codecs such as Draco and G-PCC, and learned codecs such as OctSqueeze and RIDDLE, can achieve higher compression ratios but expose only quantization or quality settings rather than a user-specified, certified geometric bound. Because this certified bound is the fidelity contract anchored on-chain by the verifiable-archival layer (Section 3.3), BEDM treats the codec as a pluggable component: a deployment that does not require certified bounds could substitute such a codec without changing the TSN or blockchain layers. This certified bound matters concretely for the targeted use case: the on-chain fidelity tier is a contractual reconstruction-error commitment—relevant to audit-grade or forensic retention—that a quantization- or quality-parameter codec cannot provide. A more recent example is the real-time neural codec RENO [27], evaluated on automotive LiDAR (KITTI/SemanticKITTI), which improves coding speed and bitrate over Draco and G-PCC but, like other learned codecs, is parameter-defined rather than bound-certified.

### 2.2. TSN in Automotive Systems

TSN is a leading deterministic communication standard for next-generation automotive Ethernet [14]. The IEEE 802.1AS standard provides sub-microsecond time synchronization through PTP, establishing a common time reference across all network devices [8]. IEEE 802.1Qbv introduces TAS, which divides time into deterministic transmission windows to enable guaranteed bandwidth allocation and bounded latency for critical sensor streams [9].

Recent automotive TSN studies demonstrate the technology’s potential for in-vehicle networks. Luo et al. [28] developed an OMNeT++-based methodology showing that TSN can better handle high-bandwidth sensor data than legacy in-vehicle networks. Zhou et al. [15] simulated TSN traffic scheduling and shaping for future automotive Ethernet and reported significant worst-case latency reductions versus non-time-aware shaping configurations. However, these investigations typically assume static, uncompressed data streams with fixed bandwidth demands.

Classical TSN scheduling algorithms—including no-wait packet scheduling [10] and SMT-based formulations [11]—optimize network utilization under strict timing constraints. Craciunas et al. [11] showed that scheduling real-time communication in IEEE 802.1Qbv requires careful consideration of stream dependencies and network topology. While these methods provide deterministic guarantees, they assume fixed stream parameters—an assumption that breaks down when compression ratios vary over time. Designing harmonized periods (common hyperperiods across streams) can simplify scheduling, but this has not been explored together with variable compression scenarios. This gap between static TSN assumptions and dynamic compression is an optimization opportunity that current architectures do not exploit. A more recent partial integration, TECChain [29], couples TSN synchronization with blockchain-based edge-to-edge collaboration management, but it does not target LiDAR data paths or error-bounded compression. A recent systematic review and experimental study by Xue et al. [30] benchmarked 17 representative IEEE 802.1Qbv scheduling methods across high-fidelity simulation and a hardware testbed and provides the open-source TSNkit toolkit used in our evaluation.

### 2.3. Blockchain and Distributed Storage for Vehicular Networks

Blockchain technology addresses trust and data-integrity requirements in vehicular networks. Ye and Park [12] integrated blockchain with IPFS for reliable vehicle data storage, showing that decentralized storage can keep data accessible without relying on a central repository. They store content identifiers (CIDs) on-chain while leveraging IPFS for payload storage, substantially reducing on-chain storage requirements. Danish et al. [13] proposed BlockEV for secure electric-vehicle charging, showing that blockchain can handle vehicle-to-infrastructure transactions with limited overhead. More recent work continues this direction: Ahmed et al. [31] combine blockchain with IPFS for vehicular ad hoc network data, recording content identifiers on-chain while storing payloads off-chain and verifying integrity through the content identifier—consistent with the off-chain-payload, on-chain-commitment pattern adopted in this paper.

IPFS [16] provides content-addressed, versioned storage that is well-suited to immutable sensor data. In vehicular contexts, IPFS enables distributed redundancy without a central repository, which is desirable for resilient ADSs. Surveys on blockchain for IoT [32] and for vehicular networks/trust management [17] report applications in areas such as authentication, secure communication, and reputation and trust management. However, most blockchain-vehicular integrations target low-bandwidth tasks (e.g., authentication, access control, or periodic status updates). The integration with high-frequency, high-volume sensor data remains largely unexplored. Existing solutions either compress data without blockchain verification or use blockchain without addressing data volume—missing the synergistic benefits of a combined design.

### 2.4. Integration Gaps and Research Motivation

While compression, TSN, and blockchain technologies individually address different aspects of data challenges in ADSs, using them in isolation leads to inefficiencies. Compression reduces data volume but is rarely network-aware; TSN provides deterministic transmission but typically assumes fixed bandwidth; blockchain ensures integrity and auditability but struggles with data scale and throughput.

Partial integration attempts show both promise and limitations. Bandwidth-aware or network-aware compression approaches adapt compression ratios based on available capacity, but they generally do not provide end-to-end deterministic guarantees across the network [33]. TSN deployments that incorporate compressed streams show promise, yet they often require manual (re)configuration as compression settings change, and even recent online TSN schedulers do not model compression-induced variability directly within the scheduler [34]. As a result, designers must sacrifice compression efficiency for predictability, compromise integrity for performance, or accept high infrastructure cost.

Our work addresses this gap with a framework that combines error-bounded compression algorithms [7] with TSN scheduling and blockchain-based storage. The three components reinforce each other: compression enables more streams within TSN transmission windows; TSN ensures timely, bounded-latency delivery for safety-critical perception; and blockchain verification benefits from compression’s strict error bounds and content addressing. By co-designing certified-error compression with deterministic networking and verifiable storage, the framework targets bandwidth efficiency, real-time performance, and data integrity together. As Table 1 shows, prior work has largely studied the main ingredients of BEDM as separate problems. Foundational bounded-error compression studies establish accuracy-controlled data reduction, LiDAR-specific work focuses on point cloud compression, TSN studies address deterministic networking, and blockchain-based work addresses trusted coordination or storage. BEDM is positioned at the intersection of these lines of work by combining bounded-error LiDAR compression, the TSN layer, and the blockchain/IPFS layer within one evaluated pipeline. We therefore present BEDM as a system-level integration and cross-layer evaluation, rather than as a new standalone codec, TSN scheduler, or blockchain protocol.

## 3. System Architecture

The BEDM framework is an IoT data engineering architecture for high-volume in-vehicle sensor data. It covers end-to-end processing, transmission, storage, and retrieval of LiDAR data for real-time autonomous driving.

### 3.1. BEDM System Overview

The framework uses a four-layer architecture to handle high-volume sensor data in autonomous driving systems. The BEDM framework organizes compression, networking, and storage into a unified pipeline covering data acquisition, transformation, deterministic delivery, and secure availability. This reflects the needs of IoT systems where sensor data must be processed reliably and with privacy guarantees before it can support downstream decisions.

The architecture begins with a data acquisition layer that ingests heterogeneous sensor streams, including LiDAR, cameras, radar, and vehicle control data. LiDAR point clouds dominate network traffic and storage consumption, so the framework targets LiDAR data while remaining extensible to other sensor modalities.

The compression layer implements our error-bounded algorithms to reduce this data volume while preserving geometric accuracy. Unlike traditional lossless compression that offers limited reduction ratios, our approach allows controlled information loss within specified bounds, enabling significantly higher compression ratios while constraining geometric reconstruction error under the configured bound. The TSN scheduling layer then ensures deterministic delivery of the compressed data through IEEE 802.1AS time synchronization and 802.1Qbv time-aware shaping. Finally, the storage and security layer provides long-term data persistence through IPFS distributed storage and blockchain-based integrity verification.

**Figure 1:**
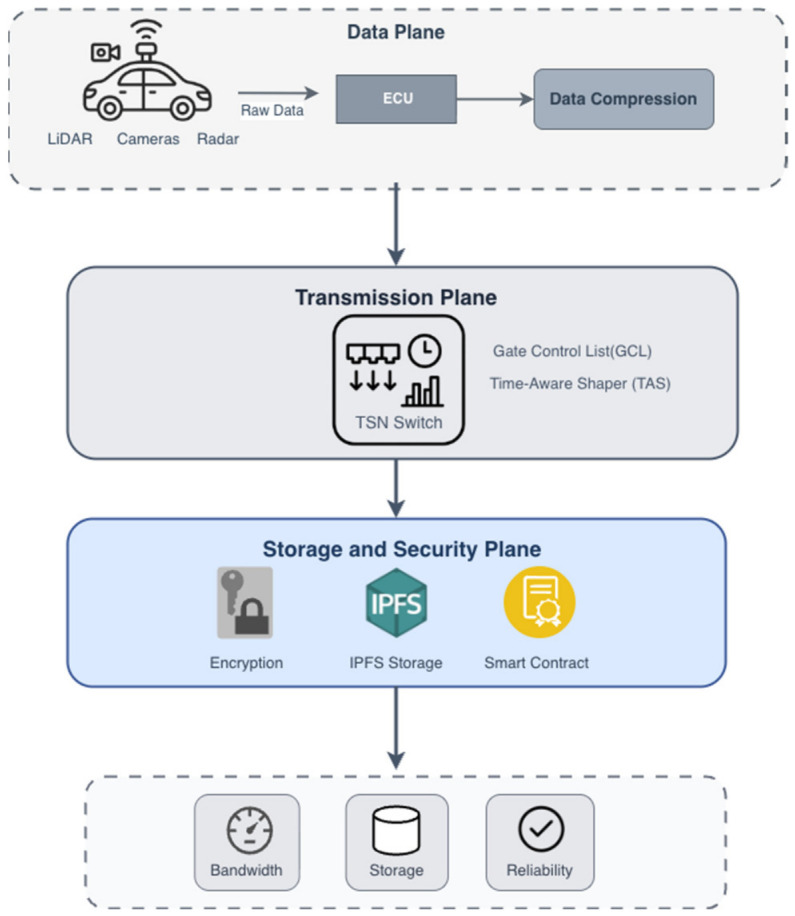
System overview.

Figure 1 illustrates our integrated framework comprising four main modules: (1) data acquisition from LiDAR and camera sensors, (2) error-bounded compression, which reduces data volume; (3) TSN scheduling, which ensures deterministic delivery; and (4) privacy-preserving storage, which combines encryption, IPFS, and blockchain verification.

### 3.2. Compression Strategies

In the BEDM framework, compression both reduces bandwidth and prepares raw LiDAR point clouds for efficient transmission, storage, and retrieval.

Our framework implements seven distinct compression methods, each offering different trade-offs between compression ratio, computational complexity, and reconstruction accuracy. The baseline Huffman coding serves as a lossless reference, achieving modest compression through frequency-based encoding but limited by the entropy of the input data. Building upon this foundation, we developed error-bounded variants that exploit the inherent redundancy in point cloud data.

EB-HC merges nearby points within specified error bounds before applying Huffman encoding. We implement two variants: Axis-aligned merging that considers each dimension independently, and L2-norm merging that uses Euclidean distance. The merging process groups points where all members lie within the specified error bound from a representative point, typically the centroid. This approach reduces the number of unique coordinates while preserving local spatial structure under the configured geometric error bound.

EB-3D leverages hierarchical spatial subdivision to achieve compression. Starting with the complete 3D bounding box, the algorithm recursively subdivides space into eight octants until all points within each leaf node satisfy the error-bound constraint. The tree structure is then encoded efficiently, with empty nodes pruned to reduce storage. Similar to EB-HC, we implement both Axis-aligned and L2-norm variants.

The hybrid approach, EB-HC-3D, combines the strengths of both octree subdivision and Huffman coding. The algorithm first applies octree partitioning to organize the spatial data, then encodes the resulting tree structure using Huffman coding. This combination exploits both spatial and statistical redundancy for higher compression ratios.

**Figure 2:**
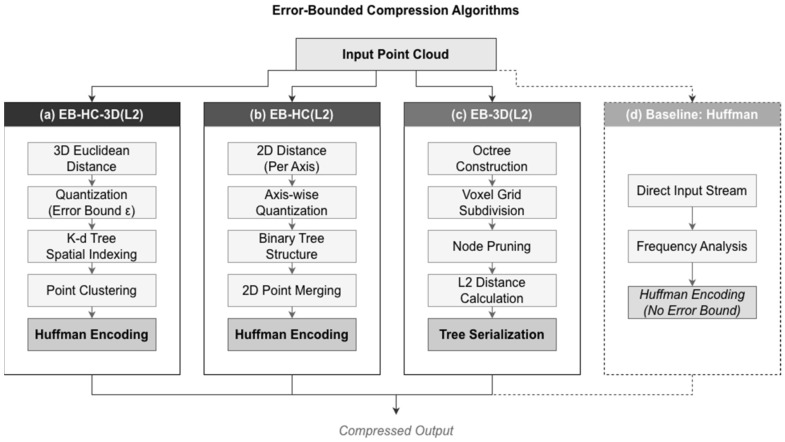
Comparison of compression algorithms.

Figure 2 compares three error-bounded compression methods by illustrating the processing pipeline, error-bound enforcement, and output characteristics. EB-HC-3D achieves the best compression by combining octree partitioning with Huffman coding. Table 2 summarizes the algorithmic characteristics of these strategies.

### 3.3. Blockchain/IPFS-Based Error-Bound-Aware Verifiable Archival Layer

The storage layer provides confidential and tamper-evident archival for compressed LiDAR records without placing blockchain operations on the real-time TSN-bounded delivery path. After error-bounded compression, each compressed frame *C_t_* is encrypted with AES-GCM-256 using canonical metadata *M_t_* as Additional Authenticated Data (AAD). The metadata records the object-schema version, vehicle identifier hash, sensor identifier, frame identifier, timestamp, compression method, declared reconstruction error bound, compressed payload size, compressed-payload hash, and previous-record identifier. The resulting IPFS object *O_t_* contains the canonical encoding of {version, *M_t_*, nonce*_t_*, *E_t_*, tag*_t_*}, where *E_t_* is the encrypted payload and tag*_t_* is the AES-GCM authentication tag. The CID returned by IPFS therefore identifies ciphertext rather than plaintext. Confidentiality is provided by AES-GCM, while IPFS provides content-addressed retrieval, and the blockchain provides tamper-evident anchoring under the stated trust and threat model.

To avoid storing high-rate sensor data on-chain, BEDM separates off-chain payload storage from on-chain verification. Each encrypted object is added to IPFS to obtain *CID_t_*, while the per-frame CID, metadata hash *h*_M_, object hash *h*_O_, and Merkle leaf are recorded in an off-chain batch manifest. Here, *h*_M_ denotes the hash of the canonical metadata and *h*_O_ denotes the hash of the encrypted IPFS object. When the batch window closes, the gateway computes a Merkle root over the batch leaves and anchors only compact verification metadata on-chain. The on-chain record includes the batch root, manifest CID hash, manifest hash, fidelity tier, declared error bound, timing fields, and record count. As a result, per-frame CIDs remain in the off-chain manifest, while the blockchain stores only batch-level commitments required for audit and verification.

The smart contract is designed to be error-bound-aware rather than a generic CID registry. It maintains a fidelity-tier registry and enforces writer authorization, declared error-bound limits, tier-specific maximum batch windows, maximum records per batch, and minimum anchoring intervals. In the evaluated configuration, the example tiers correspond to the error-bound settings used in the experiments: T0 Forensic with ε ≤ 0.5 cm, T1 Operational with ε ≤ 1.0 cm, and T2 Screening with ε ≤ 2.0 cm. These tiers allow archival frequency and declared reconstruction fidelity to be coupled in the contract policy. The contract verifies only the declared error-bound/tier policy; it does not verify the actual reconstruction error of the compressed payload, which remains a writer-side attestation produced by the vehicle-side gateway.

The verifiable archival layer is implemented as a single on-chain smart contract, BEDMAnchor, written in Solidity 0.8.20; Listing 1 discloses its external interface in condensed form, comprising its persistent state, its externally callable functions with their guard conditions, and its events, while the compiler and EVM settings used in the evaluation are given in Section 4.1. For brevity, Listing 1 omits public-visibility keywords, calldata data-location keywords, indexed event attributes, and the automatically generated getters of public state variables. The persistent state consists of the contract owner, a writer-authorization mapping with a per-writer strictness ceiling writerMinTier, the fidelity-tier registry ebTiers mapping each tierId to the policy tuple (errorBoundDmm, maxBatchWindowSec, maxRecordsPerBatch, minAnchorIntervalSec, enabled), a per-(writer, tier) last-anchoring timestamp, and the records and batches mappings that hold the anchored commitments. In addition to the automatically generated getter functions of its public state variables, the contract exposes six functions in three groups. Two owner-only administration functions, setWriter and setEBTier, manage writer authorization and tier policy; the constructor sets the deployer as owner and as a fully privileged writer (writerMinTier = 0) and initializes the three default tiers used in the experiments. Two writer-only state-mutating functions implement anchoring: storeRecord, the per-frame baseline retained for cost comparison, and anchorBatch, the recommended Merkle-batched mode invoked in line 38 of Algorithm 1, which accepts a batch only if all required guards in Listing 1 hold—batchId uniqueness (replay protection), well-formed payload and time window, tier enabled, declared error bound within the tier bound, batch window and record count within the tier limits, no writer tier escalation, and a minimum interval between consecutive anchors of the same writer and tier. Two read-only view functions support independent audit: verifyRecord checks a Merkle inclusion proof against an anchored batch root, and batchFidelity returns the anchored fidelity tier and declared error bound of a batch. Every administrative and anchoring state change emits an event (WriterSet, EBTierSet, RecordStored, BatchAnchored), so the anchoring history can be reconstructed from the event log without trusting the writer. These on-chain guards and the verifyRecord audit path are the surfaces exercised by the executable negative tests NT-3–NT-9 in Section 4.7; the remaining well-formedness, tier-enabled, record-cap, and minimum-interval guards are enforced by require checks but are not exercised as separate negative tests.


**Listing 1.** External interface of the BEDMAnchor smart contract (Solidity 0.8.20).

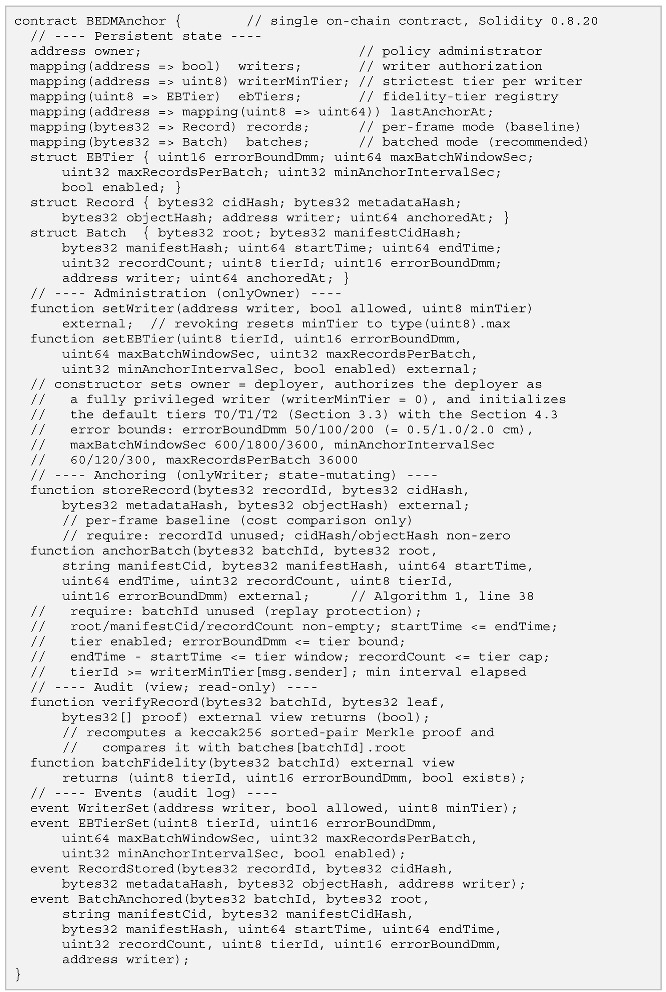


**Algorithm 1:** BEDM end-to-end data-management pipeline
**Require: schema_version; vehicle_id_hash; sensor_id; frame_id; timestamp t; previous-record identifier prev_record_id; error bound ε > 0; validation metric m ∈ {Axis, L2}; compression method method; stream configuration ψ_t_ = (src, dst, T_t_, D_t_, q_t_); MTU_payload; link rate R; active TSN stream set F; hyperperiod H; AES-GCM key K; IPFS node; address SC of the deployed BEDMAnchor contract (Listing 1).**

**Ensure: compressed payload C_t_; TSN transmission status σ_t_; IPFS content identifier CID_t_; off-chain manifest leaf leaf_t_; blockchain anchoring status/record B_batch.**
  1:   *// Data acquisition*  2:   P_t_ ← AcquireLiDARFrame(sensor_id, t)  3:   *// Error-bounded compression*  4:   C_t_ ← Compress(P_t_, ε, m)  5:   P′_t_ ← Decompress(C_t_)  6:   ok ← ValidateBound(P_t_, P′_t_, ε, m)  7:   if ok = false then  8:  σ_t_ ← NOT_ADMISSIBLE  9:  return null, σ_t_, null, null, null10:   end if11:   *// Packet-demand estimation and TSN stream construction*12:   n_t_ ← ceil(|C_t_|/MTU_payload)13:   r_t_ ← 8|C_t_|/T_t_14:   f_t_ ← (src, dst, T_t_, D_t_, q_t_, n_t_, r_t_)15:   *// TSN schedulability validation*16:   if no valid GCL exists for F ∪ {f_t_} then17:  feasible, GCL ← TSN_Schedule(F ∪ {f_t_}, H, R)18:  if feasible = false then19:   σ_t_ ← UNSCHEDULABLE20:   return C_t_, σ_t_, null, null, null21:  end if22:   else23:  GCL ← ActiveGCL24:   end if25:   *// TSN transmission*26:   packets ← Segment(C_t_, MTU_payload)27:   σ_t_ ← TSN_Transmit(packets, GCL)28:   *// Secure distributed archival*29:   M_t_ ← {schema_version, vehicle_id_hash, sensor_id, frame_id, t, ε, m, method, |C_t_|, SHA-256(C_t_), prev_record_id, σ_t_}30:   nonce_t_ ← NonceGen()31:   E_t_, tag_t_ ← AES_GCM_Encrypt(K, nonce_t_, C_t_, AAD = M_t_)32:   O_t_ ← Serialize(M_t_, nonce_t_, E_t_, tag_t_)33:   CID_t_ ← IPFS_Add(O_t_)34:   hM ← SHA-256(M_t_); hO ← SHA-256(O_t_)35:   leaf_t_ ← AppendToBatchManifest(CID_t_, hM, hO)36:   if BatchWindowClosed() then37:  batchArgs ← CloseBatchManifest()38:  B_batch ← AsyncCall(SC.anchorBatch(batchArgs))39:   else40:  B_batch ← PENDING41:   end if42:   return C_t_, σ_t_, CID_t_, leaf_t_, B_batch


BEDMAnchor conforms to the two execution constraints that the EVM imposes on smart contracts: determinism and passivity. Regarding determinism, every function in Listing 1 is a deterministic state transition computed solely from the transaction calldata and the current contract storage: the contract performs no file or network input/output, calls no other contracts or oracles, uses no randomness, and contains no floating-point arithmetic—the declared error bound is represented on-chain as the integer errorBoundDmm in units of 0.1 mm precisely so that the tier-bound check reduces to an exact integer comparison. The only execution-environment values read are msg.sender and block.timestamp, which are fixed by the transaction signature and by the consensus-agreed header of the including block, respectively, so every node that replays the transaction computes an identical state transition. Regarding passivity, the contract contains no autonomous, scheduled, or event-driven behavior: its code executes only when an externally signed transaction invokes a state-mutating function or a client issues a read-only call to a view function; in particular, the contract cannot observe batch windows or initiate anchoring by itself. All inherently active or non-deterministic steps of the pipeline—sensor acquisition, compression and bound validation, TSN scheduling and transmission, AES-GCM nonce generation, IPFS input/output, and batch-window timing—are therefore executed off-chain by the in-vehicle gateway (Section 3.5), and the blockchain serves purely as a passive, deterministic commitment registry.

The trust model is limited to archival integrity. The in-vehicle gateway is trusted at write time to generate the compressed payload, construct canonical metadata, encrypt the object, and submit the correct batch commitment. The AES-GCM key custodian and writer-key custodian are also trusted. In contrast, IPFS gateways, network paths, blockchain RPC endpoints, and parties without the authorized writer or AES keys are treated as untrusted. BEDM targets archival tampering, including ciphertext modification, metadata substitution, unauthorized anchoring, replay or duplicate anchoring, forged Merkle inclusion, and manifest substitution. It does not address LiDAR sensor spoofing, authorized writers producing semantically false records, key compromise, denial of service, smart-contract bugs outside the evaluated surface, or chain-level finality attacks.

**Figure 3:**
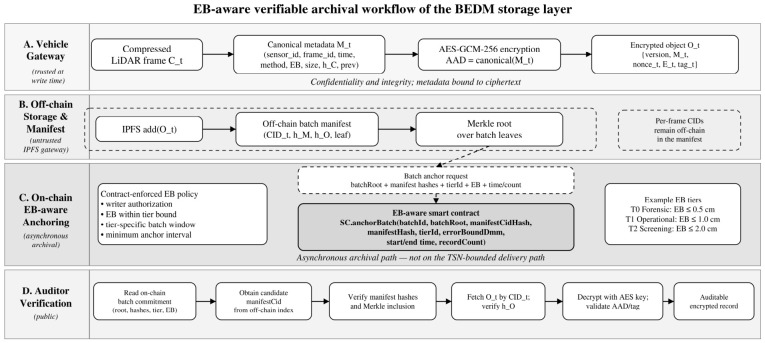
Error-bound-aware verifiable archival workflow.

Verification follows the workflow shown in Figure 3. An auditor first reads the on-chain batch commitment and obtains a candidate manifest CID from the off-chain manifest index or retrieval service. The auditor then verifies that the candidate manifest CID matches the on-chain manifest CID hash, retrieves the manifest, checks the manifest hash, and validates Merkle inclusion for the target frame. After retrieving the encrypted object from IPFS using *CID_t_*, the auditor verifies the object hash *h*_O_ and decrypts the object using the authorized AES key with canonical metadata as AAD. AES-GCM tag verification rejects ciphertext or metadata tampering, while manifest-hash and Merkle-inclusion checks reject manifest substitution and forged membership claims. Because blockchain anchoring is asynchronous, this verification path supports audit and retrieval rather than real-time control.

Figure 3 summarizes the resulting archival workflow. The compression and TSN stages remain on the real-time data-delivery path, whereas IPFS storage, manifest construction, and smart-contract anchoring form an asynchronous archival path. This design allows compressed LiDAR records to be stored and later verified without requiring blockchain confirmation in the TSN-bounded delivery loop. It also makes the error-bound policy explicit in the storage layer: the contract records not only whether a batch was anchored, but also the declared fidelity tier and error-bound metadata under which the archival commitment was made.

### 3.4. TSN Scheduler

TSN serves as the deterministic delivery layer, transmitting processed sensor data on time for downstream perception and decision-making. The system model assumes IEEE 802.1AS-compatible network-wide time synchronization, while the evaluated schedulability analysis focuses on IEEE 802.1Qbv-compatible time-aware shaping. This common time base enables coordinated schedules that prevent conflicts.

The scheduling algorithm operates on harmonized periods designed to minimize complexity while maximizing schedulability. We selected periods that result in a hyperperiod of 100 ms as the least common multiple: 100 ms for LiDAR (10 Hz sampling rate), 20 ms for front cameras (50 Hz), and other periods for remaining sensors. This harmonization simplifies scheduling and improves network utilization.

Central to our TSN implementation is the Gate Control List (GCL), which specifies precisely when each priority queue can transmit. GCL divides the hyperperiod into time slots, with each slot dedicated to specific traffic classes. Critical LiDAR data receives priority allocation in the earliest slots, followed by high-priority camera data, medium-priority radar, and finally low-priority control messages. This prioritization keeps safety-critical data within its latency budget.

### 3.5. Data Flow

**Figure 4:**
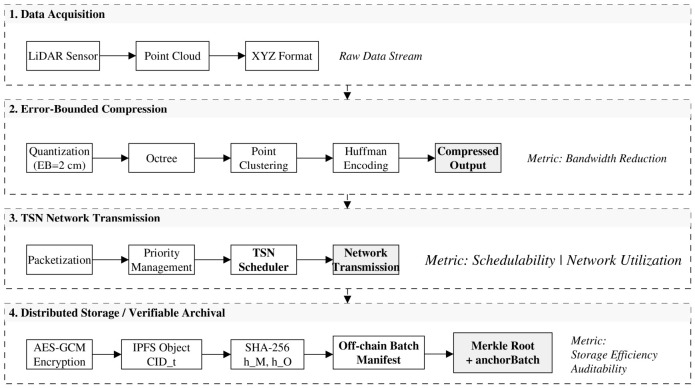
Data-flow pipeline.

Figure 4 illustrates the complete data flow through our system, showing the end-to-end pipeline from acquisition through storage. The process can be described in four logical stages: data acquisition, error-bounded compression, TSN-based transmission, and distributed storage. In Algorithm 1, the TSN-based transmission stage is further expanded into packet-demand estimation, schedulability validation, and scheduled transmission. To make the operational model of BEDM explicit, Algorithm 1 summarizes the end-to-end data-management pipeline. The algorithm is not intended to replace the internal implementation of the underlying compression, TSN scheduling, IPFS, or blockchain mechanisms; rather, it formalizes how these components are connected in the proposed cross-layer framework. In particular, Algorithm 1 is not itself implemented as a smart contract and does not execute on-chain: the entire pipeline runs off-chain on the in-vehicle gateway, while the blockchain participates only through the passive, deterministic BEDMAnchor contract disclosed in Section 3.3 (Listing 1), which the gateway invokes in line 38.

Let P_t_ denote the raw LiDAR point cloud of N_t_ points p_i_ acquired at time t. For a user-specified error bound ε, the compression stage produces a compressed representation C_t_ and the reconstructed point cloud P′_t_. For each original point p_i_, let p′_a_(i) denote its reconstructed representative. The admissibility condition is max_i_ d_m_(p_i_, p′_a_(i)) ≤ ε, where d_m_ is either the axis-aligned maximum deviation or the L2 distance. The compressed payload size |C_t_| determines the packet demand n_t_ = ceil(|C_t_|/MTU_payload), and the corresponding compressed bitrate is r_t_ = 8|C_t_|/T_t_. These quantities are mapped to the TSN stream descriptor f_t_ = (src, dst, T_t_, D_t_, q_t_, n_t_, r_t_), where T_t_ is the period, D_t_ is the deadline, q_t_ is the priority class, and H is the harmonized hyperperiod.

Algorithm 1 abstracts the runtime interaction among the compression, networking, and storage layers. The bounded-error validation step ensures that compression remains within the configured geometric tolerance before the payload is admitted to the TSN transmission path. The packet-demand estimate connects the compressed payload size to the TSN stream model, allowing the scheduler to evaluate whether the resulting traffic set remains feasible over the harmonized hyperperiod. After TSN transmission, the compressed payload is encrypted and stored off-chain in IPFS, while the per-frame CID, metadata hash, object hash, and Merkle leaf are recorded in an off-chain batch manifest. If the batch window is not yet closed, the record remains pending in the manifest; when the batch window closes, the gateway derives batchId, root, manifestCid, manifestHash, startTime, endTime, recordCount, tierId, and errorBoundDmm from the closed batch manifest, tier policy, and time window and submits them as the arguments of anchorBatch; the contract recomputes manifestCidHash = keccak256(manifestCid) on-chain, so per-frame CIDs remain off-chain and contract storage holds only the batch root and the manifest-level commitment hashes. The variable errorBoundDmm stores the declared error bound in deci-millimeters (0.1 mm) as an integer fixed-point value. Blockchain confirmation is therefore treated as an asynchronous archival step and is not placed on the real-time control path. Of the operations in Algorithm 1, only the call SC.anchorBatch in line 38 executes on-chain: the gateway signs and submits the transaction, and the contract passively applies the deterministic require guards and storage writes disclosed in Listing 1.

First, in the data acquisition stage, the LiDAR sensor completes a scan, converting raw signals into a standard XYZ point cloud format. This raw data stream is then passed to the error-bounded compression stage. In the evaluated configuration, EB-HC-3D processes the data through quantization (2 cm error bound), octree construction, point clustering, and Huffman encoding to generate the compressed output.

Subsequently, in the TSN network transmission stage, the compressed data is segmented into packets. The priority management module assigns traffic classes according to GCL, ensuring that the TSN scheduler grants high-priority access for deterministic transmission over the network.

Finally, the data enters the distributed storage stage for secure archiving. The data first undergoes AES-GCM-256 encryption for confidentiality, followed by IPFS chunking and SHA-256 hashing to generate unique CIDs. While the encrypted chunks are distributed across the IPFS P2P network, the resulting CID is recorded in an off-chain batch manifest, whereas the blockchain stores only the batch-level commitment and verification hashes via a smart contract. By anchoring only batch-level commitments on-chain, the framework reduces blockchain overhead while preserving a verifiable reference for future data retrieval, integrity checking, and controlled sharing across distributed vehicular or IoT environments.

## 4. Implementation and Experiment

### 4.1. Experimental Setup

Our experimental evaluation utilized the KITTI Vision Benchmark Suite, a widely used dataset for autonomous driving research. We selected 1140 LiDAR point cloud frames from the KITTI raw data, covering five scene categories: city, residential, road, campus, and person. Each scan provides a 360-degree Velodyne point cloud stored as binary floating-point values containing x, y, z coordinates and a reflectance value, with about 100,000–120,000 points per frame.

**Figure 5:**
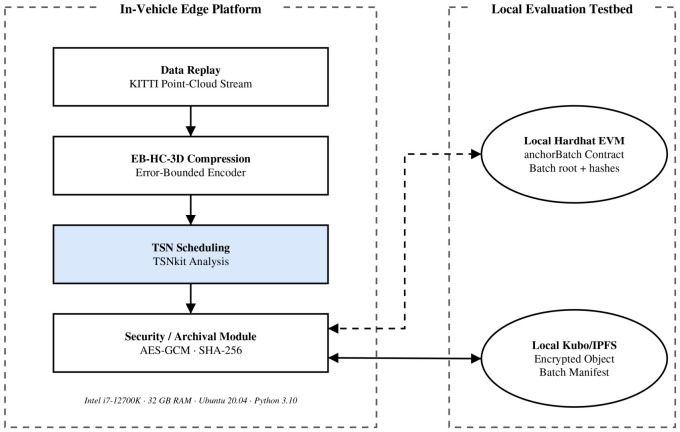
Overall evaluation architecture of the proposed BEDM framework.

The evaluation platform was an Intel Core i7-12700K (Intel, Santa Clara, CA, USA) workstation operating at 3.8 GHz with 32 GB of DDR4 memory. We treat this as a desktop-class edge/development platform that serves as a proxy evaluation environment. Two evaluation paths are reported, and we keep them clearly separated throughout this paper. First, the compression pipeline and the storage pipeline were exercised as direct measurements on this workstation: the error-bounded compression methods were run locally over the KITTI data, and IPFS ingestion was performed against a local Kubo/go-IPFS 0.7.0 node using default chunking, sha2-256 content addressing, and local pinning; distributed multi-node replication and availability were not evaluated, and the storage path produced the IPFS latency and wrapper-overhead results reported in Section 4.7. The contract path measures gas costs and negative-test outcomes against the fidelity-aware BEDMAnchor contract in Section 3.3 (Listing 1), deployed on a local Hardhat EVM (chainId 31337), which uses the same gas accounting as Ethereum mainnet but provides deterministic, reproducible measurements. This is the path that produced the gas and negative-test results reported in Section 4.7. The two paths are kept separate because they answer different questions: “is the compressed-payload storage path fast enough” versus “is the verifiable-archival contract sound and how much gas does it consume on the canonical EVM”. The anchor contract was implemented in Solidity 0.8.20, with the optimizer enabled at 200 runs and evmVersion = paris. AES-GCM-256 was used with a fresh 96-bit nonce per frame and canonical JSON-encoded metadata bound as AAD. IPFS objects were added with cid-version=1 and hash=sha2-256. The end-to-end PoC reported in Section 4.7 exercises the full encrypt + IPFS-add + anchor + verify path against the same local kubo daemon and contract used above; no live mainnet or testnet anchoring was claimed in the evaluation. Second, TSN behavior was evaluated through IEEE 802.1Qbv-compatible TAS schedulability analysis using TSNkit 0.2.0; IEEE 802.1AS time synchronization is assumed as part of the system model rather than implemented in the TSNkit evaluation. TSNkit provides scheduling and benchmarking algorithms, including list-scheduling, SMT-based formulations, and heuristic methods. The reported TSN latency and link-utilization numbers therefore come from TSNkit under the specified flow set and topology. Figure 5 shows the overall experimental architecture combining these two evaluation paths.

The software environment was built on Ubuntu 20.04 LTS with Python 3.10 for algorithm implementation and data processing. TSN scheduling used TSNkit version 0.2.0 as a scheduling and benchmarking toolkit, with list-scheduling, SMT-based approaches, and heuristic methods. All compression algorithms were implemented in Python with NumPy 2.2.6 optimizations for vectorized operations, supporting prototype-level timing evaluation on the desktop-class edge/development platform. The TSNkit toolkit and the 802.1Qbv scheduler benchmark it builds on are described by Xue et al. [30].

### 4.2. Compression Performance Evaluation

**Figure 6:**
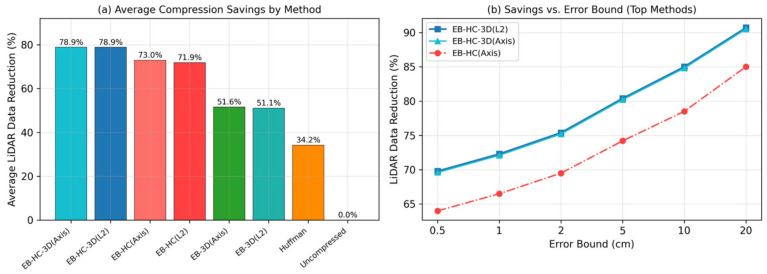
Compression efficiency analysis: (**a**) average bandwidth savings of the seven compression methods across all tested error bounds; (**b**) compression savings versus error bound for the top-performing EB-HC and EB-HC-3D methods. Per-method statistical measures (per-frame 95% confidence intervals and a one-way ANOVA) are reported in Section 4.2.

The evaluation of the seven compression strategies revealed a distinct performance hierarchy, as summarized in Figure 6. Figure 6a illustrates the average bandwidth savings across all tested error bounds. Our EB-HC-3D methods achieved the highest efficiency, with both Axis-aligned and L2-norm variants delivering average savings of 78.9%. In comparison, the EB-HC methods averaged 73.0% (Axis) and 71.9% (L2), while EB-3D lagged behind at approximately 51%. The baseline Huffman coding, while lossless, provided only a 34.2% average reduction, which is insufficient for the high-bandwidth demands of ADSs.

Figure 6b details the relationship between the error bound and compression savings for the top-performing methods. A distinct pattern emerges, where efficiency improves with the error bound but with diminishing marginal returns. The EB-HC-3D(Axis) and EB-HC-3D(L2) variants show identical bandwidth savings across all measured points, resulting in overlapping curves in the figure; this indicates that the choice of distance metric (Axis vs. L2) in the 3D hybrid approach did not significantly alter the final entropy of the compressed stream in our dataset.

**Figure 7:**
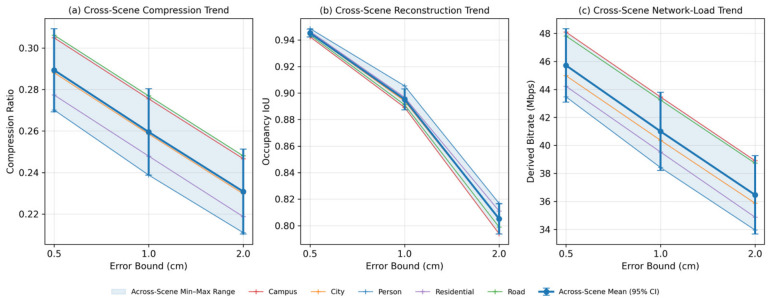
Cross-scene sensitivity of EB-HC-3D(L2) across the five KITTI scene groups (solid line: across-scene mean; shaded band: min–max range). Error bars on the across-scene mean denote 95% confidence intervals across the five scenes.

**Figure 8:**
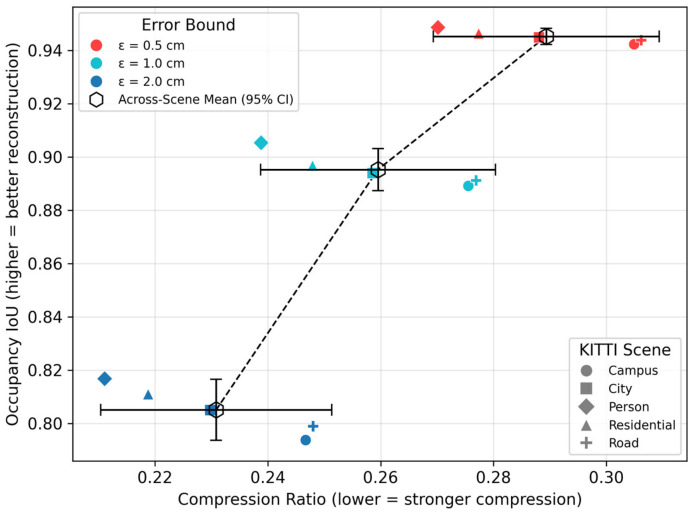
Compression–reconstruction trade-off of EB-HC-3D(L2) across five KITTI scenes and three error-bound settings (0.5, 1.0, 2.0 cm). Error bars on the across-scene mean markers denote 95% confidence intervals across the five scenes.

To quantify the statistical reliability of these results, we computed per-frame statistics over the evaluation set (389 frames per method and error bound; the same set used for the storage-overhead analysis in Section 4.7). Per-frame bandwidth savings are tightly distributed: the 95% confidence-interval half-width was at most 0.16 percentage points for every method, indicating low sampling variability. A one-way ANOVA across the seven methods at ε = 2 cm confirmed that the differences among methods were highly significant (F = 8.9 × 10^4^, *p* < 10^−3^), and the EB-HC-3D advantage over EB-HC corresponded to a very large effect size (Cohen’s d = 7.16). The Axis-aligned and L2-norm variants of EB-HC-3D differed by less than 0.001 percentage points, consistent with the overlapping curves shown in Figure 6b. Per-error-bound cross-scene confidence intervals are reported in Table 3 and Figure 7 and Figure 8.

We selected a practical operating point at an error bound of 2 cm using an explicit fidelity-constrained criterion: 2 cm was the largest error bound for which the across-scene mean Occupancy IoU remained at or above an illustrative geometric-fidelity floor of 0.80 (0.805 at 2 cm); beyond 2 cm, the mean Occupancy IoU fell steeply (0.607 at 5 cm and 0.131 at 20 cm in a corpus-wide error-bound sweep) in exchange for only marginal additional bandwidth savings. The 0.80 floor is a transparent, reproducible selection convention rather than a perception-certified threshold—indeed, 0.80 lies within the across-scene confidence interval around the measured 0.805 (Table 3), so the two are not statistically distinguishable—and 2 cm should therefore be considered a representative operating point whose perception-level adequacy is left to future work, as noted in Section 5. At this configuration, the EB-HC-3D algorithm achieved a 75.4% reduction in LiDAR data volume in the full method-comparison aggregation used for Figure 6. This efficiency reduced the total system data rate to 204.94 Mbps (utilizing only 20.49% of network capacity). This was an improvementd on the EB-HC(Axis) method, which achieved 69.5% savings (resulting in 223.54 Mbps) at the same error bound.

Although increasing the error bound to 20 cm further boosted savings to 90.7% (156.71 Mbps), the marginal gains diminished beyond 5 cm, while the reported reconstruction-quality metrics degraded. Therefore, the 2 cm configuration was selected as a practical operating point in this evaluation, providing substantial compression while maintaining a mean reconstruction error of 0.87 cm under the reported geometric metric.

### 4.3. Cross-Scene Sensitivity Analysis

To complement the method comparison across error bounds, we additionally report a supplementary cross-scene sensitivity analysis of EB-HC-3D(L2) within the currently available real dataset. Figure 7 visualizes the scene-wise trends by plotting the across-scene mean together with the min–max range at each error-bound setting. Table 3 summarizes the across-scene mean values at each error-bound setting. The analysis is based on the measured compression summary, reorganized into five KITTI scene groups (campus, city, person, residential, and road). Here, the Occupancy IoU is the intersection-over-union between the sets of occupied voxels of the original and reconstructed point clouds (jointly occupied voxels divided by the union of occupied voxels); a value of 1.0 denotes identical occupancy and lower values indicate greater reconstruction distortion. Across scenes, the across-scene mean compression ratio was 0.2893 at error bounds of 0.5 cm, 0.2595 at 1.0 cm, and 0.2309 at 2.0 cm, while the corresponding Occupancy IoU decreased from 0.9452 to 0.8953 and 0.8051. For the 1.0 cm reference case, the per-scene compression ratio ranged from 0.2388 to 0.2769, the Occupancy IoU ranged from 0.8892 to 0.9054, and the derived per-stream bitrate ranged from 38.42 to 43.46 Mbps, corresponding to a derived link utilization of 3.84% to 4.35% on the 1 Gbps link. Scene variation therefore affected both compression ratio and reconstruction quality, but the derived bitrate remained low and stable within the evaluated setup, and the main conclusion that the 2 cm operating point offers a practical trade-off is not affected by scene variation. The values in Table 3 are unweighted across-scene means, whereas the 75.4% savings reported in Section 4.2 are computed from the full method-comparison aggregation used in Figure 6; the two numbers therefore summarize different aggregation levels.

### 4.4. Compression–Reconstruction Trade-Off

To make the trade-off between compression strength, error bound, and reconstruction quality explicit, Figure 8 summarizes the same 15 (scene, ε) observations as a Pareto-style scatter, in which each point corresponds to one (scene, ε) combination, color encodes the error bound, marker shape encodes the scene, and the dashed line joins the across-scene mean at each error-bound level. Moving from ε = 0.5 cm to ε = 2.0 cm reduced the average compression ratio from 0.289 to 0.231, while the Occupancy IoU decreased from 0.945 to 0.805, with ε = 1.0 cm (0.260, 0.895) serving as an intermediate reference point between higher-fidelity and higher-compression settings.

As an independent geometric quality measure, the Chamfer distance corroborated the Occupancy IoU trend. We report the mean bidirectional squared Chamfer distance (in m^2^): it increased monotonically with the error bound (1.25 × 10^−5^, 5.07 × 10^−5^, and 2.05 × 10^−4^ m^2^ at ε = 0.5, 1.0, and 2.0 cm, corresponding to a root-mean Chamfer displacement of about 0.35, 0.71, and 1.43 cm, respectively, i.e., within the declared error bound), indicating a graceful and predictable degradation of reconstruction fidelity. Because 2 cm is small relative to the physical scale of vehicles and pedestrians, this degradation is geometrically modest.

Beyond the global error-bound trade-off, the scatter also exposes scene-level heterogeneity that the per-metric line plots cannot show directly. At every error-bound level in this dataset subset, the person scene lies on the favorable end of the compression–reconstruction trade-off—it achieves simultaneously the smallest compression ratio and the highest Occupancy IoU (0.270/0.949 at ε = 0.5 cm, 0.239/0.905 at ε = 1.0 cm, 0.211/0.817 at ε = 2.0 cm). This is consistent with the sparser and more regular geometry of person-centric scans, which EB-HC-3D(L2) can both compress more aggressively and reconstruct more faithfully. Scene-to-scene variation within each error-bound cluster is noticeably smaller than the effect of changing ε, confirming that ε remains the dominant knob for tuning this trade-off within the evaluated dataset.

### 4.5. TSN Scheduling Performance

**Figure 9:**
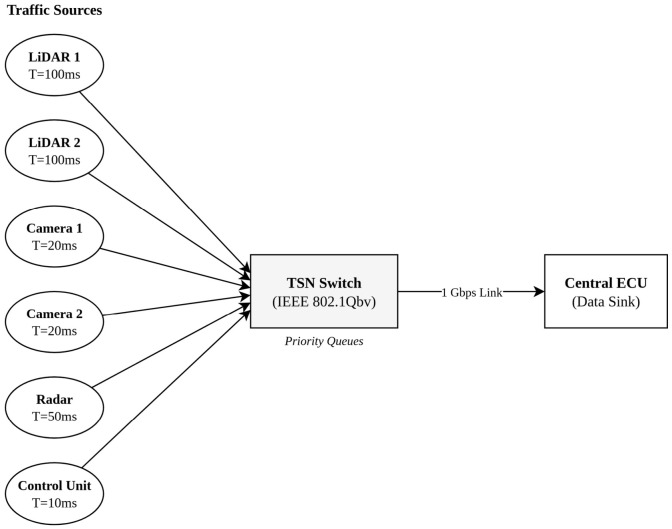
Network topology and traffic configuration for the TSN evaluation. The 8-node star topology connects six sensor nodes with varying transmission periods to a central ECU via a TSN switch over 1 Gbps links.

The TSN evaluation shows the importance of proper network design for ADSs. We simulated an eight-node star topology, consisting of six sensor nodes (two LiDARs, two cameras, one radar, and one control unit) connected to a central TSN switch, which aggregates traffic to a single electronic control unit (ECU) via 1 Gbps links. The traffic model comprised six periodic streams with harmonized periods (10 ms, 20 ms, 50 ms, and 100 ms), resulting in a common hyperperiod of 100 ms. Specifically, the high-bandwidth LiDAR tasks operate at 100 ms intervals, while critical control messages (100 bytes) operate at 10 ms intervals. Figure 9 shows the network topology and traffic configuration used for the TSN evaluation.

Using the list-scheduling algorithm in TSNkit, we successfully generated valid schedules for all compression configurations tested. The algorithm completed scheduling computation in under 500 ms, which suggests feasibility for offline or periodic reconfiguration within the evaluated setup; closed-loop online reconfiguration would require additional engineering effort and is left to future work. The resulting schedules achieved an average latency of 0.625 ms across all flows, and a maximum worst-case latency below 10 ms, indicating that all modeled streams met their assigned timing constraints in the evaluated setup.

To verify that this schedule was not an artifact of the list-scheduling heuristic, we additionally scheduled the same six-stream flow set with a representative SMT-based exact scheduler (following the formulation of Craciunas et al. [11]), adopting the benchmarking methodology of Xue et al. [30]. As summarized in Table 4, both the no-wait list scheduler [10] used in the paper and the SMT-based exact scheduler [11] produced feasible schedules that met every deadline at the same 20.49% link utilization, with solver times below 0.1 s. Because both schedulers yielded feasible schedules that met every per-flow deadline, the comparison reports schedulability, solver time, and the (identical) link utilization—the axes on which the schedulers actually differed—rather than absolute end-to-end latency: among the feasible schedules, deadline satisfaction is the guarantee of interest, and absolute latency within this schedulability-analysis abstraction is sensitive to modeling assumptions, so we report only the by-construction latency ordering and the relative hop-count effect. The two schedulers differ only in objective: the list scheduler is latency-aware—it transmits each frame as soon as possible and therefore attains the lowest end-to-end latency—whereas the SMT formulation optimizes work-conserving feasibility rather than latency, so its in-window latency is higher by construction although still within every deadline; the list scheduler is consequently adopted for the latency-critical streams. We further evaluated a two-switch cascaded topology in which every flow traverses two switch hops: both schedulers remained schedulable, and the list scheduler’s average end-to-end latency increased by roughly a factor of 2.3 relative to the single-switch case—consistent with the additional hop—while its worst-case latency stayed well within the per-flow deadlines. These results indicate that the deterministic-delivery guarantees are robust to the choice of scheduler and extend to a multi-hop in-vehicle topology, although richer ring and multi-domain topologies remain subjects of future work.

**Figure 10:**
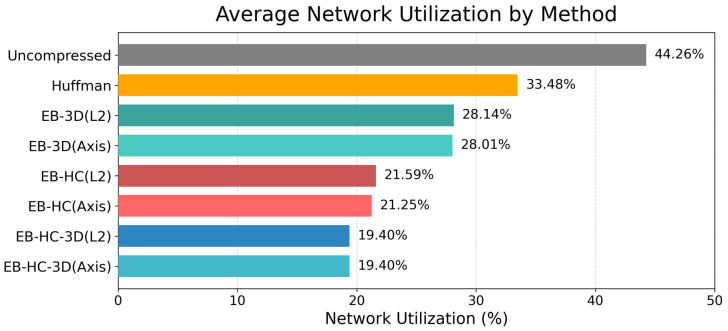
Average network utilization by compression method.

Figure 10 illustrates the average network utilization across different compression methods. The uncompressed configuration consumed 44.26% of available bandwidth (442.6 Mbps). In contrast, our proposed EB-HC-3D methods achieved the lowest average network utilization at 19.40%, with identical performance for both Axis-aligned and L2-norm variants. The baseline Huffman coding offered only a moderate reduction, to 33.48%.

Focusing on the practical operating point identified in Section 4.2 (error bound = 2 cm), the EB-HC-3D method resulted in a network utilization of 20.49% (204.94 Mbps), corresponding to a 53.7% system-wide bandwidth reduction relative to the 442.6 Mbps uncompressed baseline. This reduction from the uncompressed baseline frees bandwidth for additional sensors or multi-vehicle communication without infrastructure upgrades. In the evaluated schedule, the GCL isolates these streams by assigning dedicated transmission windows, avoiding modeled scheduling conflicts within the specified topology.

### 4.6. Schedulability Under Added Background Traffic

**Figure 11:**
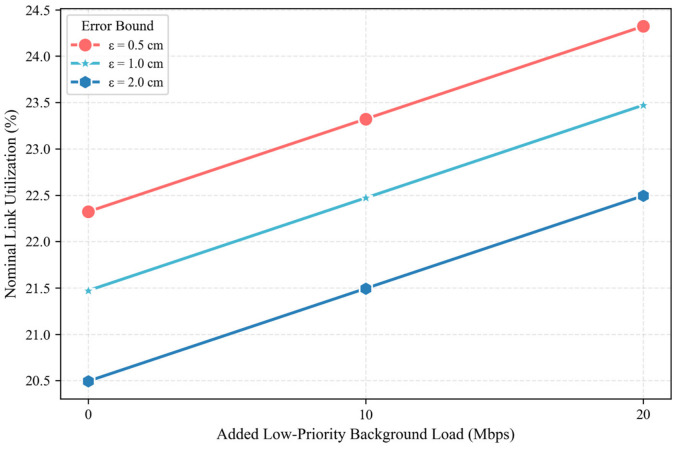
Nominal link utilization of the EB-HC-3D(L2) flow set under added low-priority background traffic (0, 10, 20 Mbps) at ε = 0.5/1.0/2.0 cm.

To examine robustness under moderate background traffic within the current packet-normalized single-switch abstraction, we augmented the existing EB-HC-3D(L2) flow sets with one additional low-priority periodic background stream (20 Hz) at 0, 10, and 20 Mbps, and reran the real TSNkit ls scheduler across ε = 0.5, 1.0, and 2.0 cm. All nine (error-bound, background-load) configurations remained schedulable. The corresponding nominal link utilization, as shown in Figure 11, ranged from 20.49% (ε = 2.0 cm, no background) to 24.32% (ε = 0.5 cm, +20 Mbps background), remaining well below the 1 Gbps link capacity in the evaluated abstraction. Because the three error-bound settings produced identical packet-level scheduling representations under this abstraction, latency-level distinctions across error bounds are not reported; instead, the supplementary analysis focuses on schedulability and link-utilization robustness.

The background loads examined here were intentionally modest relative to the 1 Gbps link; at the 2 cm operating point, the complete sensor set occupied only 20.49% of link capacity, leaving roughly 795 Mbps of nominal headroom.

### 4.7. Storage and Verifiable Archival Evaluation

This section evaluates the storage and verifiable archival path introduced in Section 3.3. The evaluation focuses on three questions: whether compressed LiDAR records can be added to the local IPFS storage path with bounded prototype latency, how much storage overhead is introduced by the AES/IPFS wrapper, and how much gas and verification costs are required for the on-chain archival functions. All blockchain measurements were performed on the local Hardhat EVM described in Section 4.1; they support reproducible gas comparison but do not measure live public-chain fees, confirmation latency, or finality.

**Figure 12:**
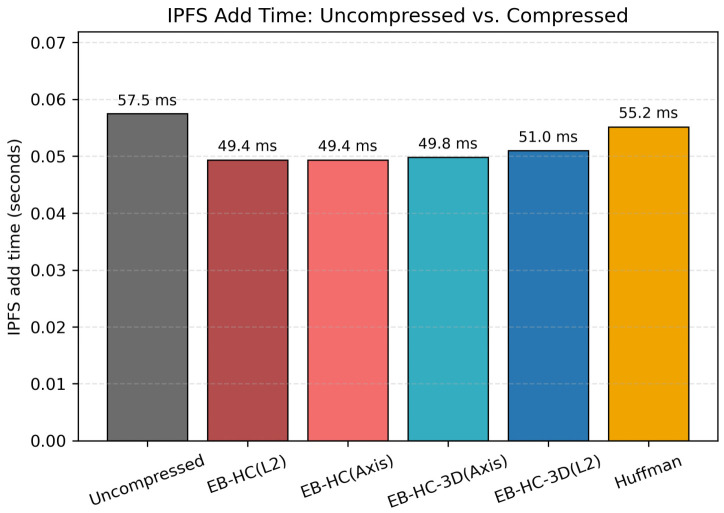
Comparison of IPFS add times: uncompressed vs. compressed.

We first evaluated the storage-side performance of the archival path by measuring local IPFS ingestion latency, defined as the time required to add and hash files on the local IPFS node, using real KITTI point cloud data. Figure 12 illustrates the processing time for adding frames to the local IPFS node.

The storage operation was dominated by IPFS implementation overheads, such as SHA-256 hashing and local database indexing, rather than raw disk I/O. Uncompressed frames required an average of 57.5 ms to process, whereas EB-HC-3D(L2)-compressed frames required an average of 51 ms. Although compression reduced the file size by more than 75%, the latency reduction was approximately 11.3%, suggesting that for sub-megabyte objects in this setup, the fixed overhead of IPFS establishes a latency floor of approximately 50 ms per file.

We next quantified the storage overhead introduced by the AES/IPFS wrapper on top of the already-compressed EB-HC-3D(L2) payload. We report the 389-frame corpus-level overhead as the primary storage-size result and use one separately selected large-payload compressed object only as a byte-level implementation check. In that check, the full wrapper, including AES-GCM-256 encryption, canonical JSON-envelope construction, and base64 ciphertext encoding, was executed end-to-end on a 1,217,711-byte EB-HC-3D(L2)-compressed payload, producing a 1,624,144-byte IPFS object and matching the wrapper specification to within 19 bytes (0.0012%). This validation object is not used as a corpus-level frame-size statistic; it verifies the byte accounting of the JSON envelope and AES-GCM wrapper. In this JSON-envelope implementation, the expansion is dominated by base64 encoding of the ciphertext plus a small fixed JSON envelope; AES-GCM itself adds only the nonce and authentication tag.

To assess whether this overhead persisted across the evaluation data, we applied the same wrapper specification to the EB-HC-3D(L2) storage-overhead subset, which contains 389 KITTI frames per error-bound setting. The AES/IPFS wrapper added mean overheads of 33.43%, 33.44%, and 33.45% relative to the EB-HC-3D(L2)-compressed payload at ε = 0.5, 1.0, and 2.0 cm, respectively. The overhead is effectively invariant across fidelity tiers because it is dominated by base64 expansion rather than by the compressor. The AES-GCM crypto-only overhead was below 0.01%, consisting of a 12-byte nonce and a 16-byte GCM tag. Replacing the JSON/base64 envelope with a binary DAG-CBOR or raw-block layout would substantially reduce serialization overhead, leaving primarily metadata, the nonce, the authentication tag, and block-encoding overhead.

**Figure 13:**
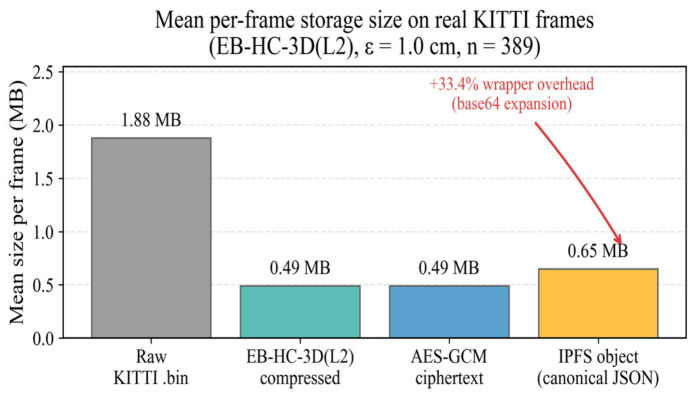
Mean per-frame size breakdown over the 389-frame Operational-tier subset (ε = 1.0 cm), showing raw → compressed → AES-GCM ciphertext → IPFS object.

The per-error-bound compression ratios of 0.2899, 0.2601, and 0.2314 obtained in this storage-overhead subset matched the Table 3 values of 0.2893, 0.2595, and 0.2309 to within 0.3%. This confirms that the wrapper analysis applies directly to the manuscript’s EB-HC-3D(L2) results and not to a substitute compressor. The 389-frame set used for storage-overhead analysis is an evaluation subset drawn from the 1140-frame compression corpus introduced in Section 4.1. The 1,217,711-byte wrapper-validation payload and the approximately 1.16 MB PoC payload reported below are the same separately selected large-payload implementation-check object, not part of the 389-frame Operational-tier storage-overhead subset used for the mean-size plot. The corpus-level storage-size claims in Figure 13 and the compression-ratio comparison were therefore based on the 389-frame subset, not on the PoC payload. The storage-overhead subset was taken from the available EB-HC-3D(L2) compression-summary rows, with the same frame rows used across error-bound settings where present; no additional random resampling was used for the wrapper statistics.

Figure 13 plots the mean per-frame size breakdown over the 389-frame Operational-tier subset (ε = 1.0 cm), from 1.88 MB raw KITTI .bin files to 0.49 MB EB-HC-3D(L2)-compressed payloads, 0.49 MB AES-GCM ciphertext, and 0.65 MB IPFS objects.

After evaluating the off-chain storage path, we measured the gas cost of the on-chain archival functions to quantify the cost of error-bound-aware batch anchoring. The gas costs of the hot-path functions of the fidelity-aware contract were measured on the Hardhat EVM, which follows the canonical EVM gas model for opcode and storage accounting. These measurements support reproducible gas comparison but do not measure live public-chain fees, confirmation latency, or finality. Across *n* = 100 calls per hot path, the recommended anchorBatch mode costs 173,965 gas (std 8), whereas the per-frame storeRecord baseline costs 117,041 gas (std 8). The 95% confidence interval on the mean was within ±2 gas in both cases, so the measurements are effectively deterministic for comparison purposes. One-time deployment costs 1,375,167 gas (*n* = 5), while administrative setWriter and setEBTier operations cost 70,571 and 51,375 gas, respectively (*n* = 30 each).

**Figure 14:**
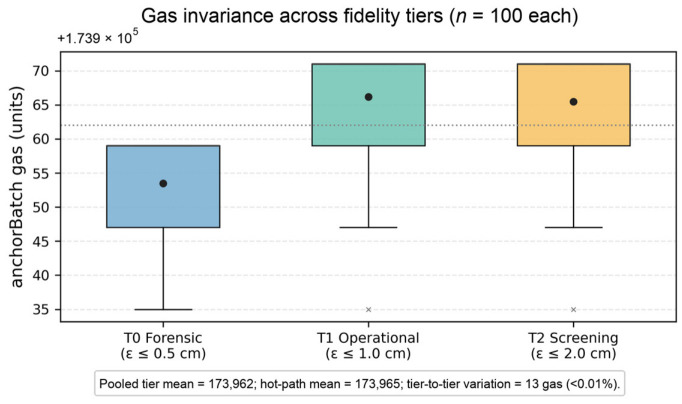
Per-tier anchorBatch gas distribution (*n* = 100 each) for the three default fidelity tiers; the near-overlapping distributions confirm that gas is structurally invariant under the fidelity-aware policy.

Figure 14 shows the per-tier anchorBatch gas distribution under each of the three default fidelity tiers. The three distributions overlap almost completely, with a mean-to-mean range of 13 gas (<0.01%). The pooled mean across the three tier-specific distributions is 173,962 gas, whereas the 173,965 gas value reported above is the separate 100-call anchorBatch hot-path mean; the 3-gas difference is rounding-level and does not affect the comparison. This confirms that anchorBatch gas is structurally invariant under the fidelity-aware policy: tier-specific behavior is enforced through require-checks rather than tier-dependent storage paths. Gas is also effectively constant in the number of records per batch because the on-chain payload consists of fixed-size commitments and metadata fields.

The per-frame storeRecord baseline and the recommended anchorBatch mode illustrate the cost advantage of batching. At a 10 Hz LiDAR rate, per-frame L1 anchoring using storeRecord would require approximately 3.69 × 10^13^ gas per vehicle per year, computed as 315 million anchors multiplied by 117,041 gas. If each frame were instead anchored as a single-record batch via anchorBatch, the annual gas requirement would be approximately 5.48 × 10^13^ gas. Both per-frame variants are financially impractical at the sensor rate under ordinary public-chain fee regimes and are retained only as comparison baselines; the recommended deployment is multi-record batch anchoring.

**Figure 15:**
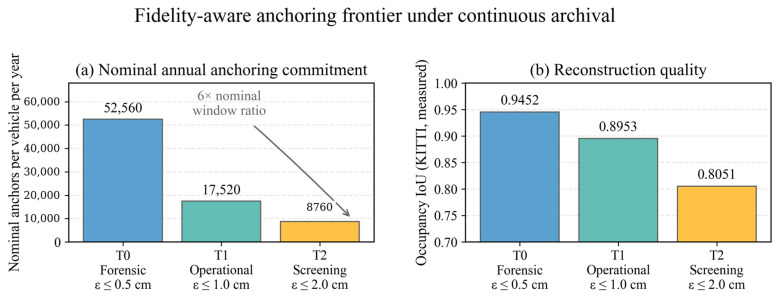
Fidelity-aware anchoring frontier under continuous archival: nominal annual anchoring commitment and KITTI Occupancy IoU across the three default fidelity tiers.

Under continuous archival and the configured tier windows, the policy implies nominal annual anchoring counts for each fidelity class. Combined with the KITTI Occupancy IoU results from Section 4.3, this yields the cross-layer frontier shown in Figure 15, linking data fidelity, reconstruction quality, and on-chain anchoring commitment. Across the three default tiers, T0 Forensic (ε ≤ 0.5 cm), T1 Operational (ε ≤ 1.0 cm), and T2 Screening (ε ≤ 2.0 cm), the nominal annual counts are 52,560, 17,520, and 8760 anchors per vehicle per year, respectively, against KITTI Occupancy-IoU values of 0.945, 0.895, and 0.805. The corresponding nominal annual gas commitments are 9.14 × 10^9^, 3.05 × 10^9^, and 1.52 × 10^9^ gas. The contract enforces the declared tier/window constraints for submitted batches, but it does not guarantee liveness or force submissions. The 6× ratio between the forensic and screening tiers is therefore a nominal consequence of the configured tier-window policy under continuous archival; USD-denominated cost remains deployment- and market-dependent.

Because USD-denominated anchoring cost depends on the deployment-day ETH price and gas-price regime, gas is reported as the primary reproducible metric. Under the illustrative fee snapshot considered here, deploying the same contract on an EVM-compatible Layer-2 network may reduce the per-anchor cost relative to L1; however, actual savings depend on the selected network, fee market, and deployment date. L1 settlement may be appropriate for low-frequency audit-grade anchoring, such as incident-triggered or hourly batches, whereas routine archival is better matched to batched or Layer-2 deployment. For submitted batches, the contract enforces the declared error bound and writer authorization, but it does not verify the actual reconstruction error of the compressed payload, which remains a writer-side attestation.

Finally, we evaluated the verification path by pairing each in-scope threat-model claim with an executable negative test. Each test ran an explicit attack scenario against either the evaluated contract or the AES-GCM-protected IPFS object and checked whether the corresponding defense was triggered. Table 5 summarizes the nine executable negative tests; all nine passed in the evaluated Hardhat contract setup. Out-of-scope threats, including availability failures, key custody compromise, chain-level finality attacks, and sensor-level attestation, require orthogonal mechanisms and were deliberately not exercised here.

NT-7 through NT-9 are specific to the fidelity-aware policy described in Section 3.3 and have no analogue in prior blockchain/IPFS archival designs without a tier policy.

The end-to-end PoC was then used to exercise the complete encrypt–IPFS-add–anchor–retrieve–decrypt–verify path. It was timed on 100 logical archival records generated from the separately selected 1,217,711-byte EB-HC-3D(L2) validation payload; this PoC does not sample 100 distinct KITTI frames. Each logical record used a distinct nonce and metadata, but the compressed byte string was the same large-payload implementation-check object. This PoC is used only to evaluate the full archival and verification path and is not used to recompute the compression-ratio statistics reported in Section 4.2, Section 4.3 and Section 4.4. The measured per-stage wall-clock means were 48.1 ms per record for encryption plus IPFS add, 16 ms per batch for manifest IPFS add, less than 12 ms for anchorBatch mining under Hardhat instant-mining mode, and 39 ms per record for independent verifier execution, including AES-GCM decryption of the approximately 1.16 MB payload. The blockchain anchoring path is asynchronous and is not part of the TSN-bounded delivery path measured in Section 4.6.

The anchorBatch mining time corresponds to Hardhat instant-mining mode and does not represent public-chain finality. Public L1/L2 confirmation latency is network-dependent and was not measured in this evaluation. The verifier time is dominated by AES-GCM decryption, whereas the vehicle-side gateway cost is represented by the encryption-plus-IPFS-add stage. Figure 12 reports only the local IPFS-add component, whereas the PoC measurement evaluates the complete archival path, including encryption, IPFS add, anchoring, retrieval, decryption, and verification. The 48.1 ms PoC value was measured on the separate 100-record large-payload archival check described above and includes a different object-size distribution from the Figure 12 per-method IPFS-add benchmark; therefore, the two measurements are complementary rather than directly comparable.

### 4.8. System Integration Results

End-to-end testing confirmed that the integrated pipeline supports steady-state pipelined ingestion within the evaluated prototype. Summing the measured stage latencies yielded approximately 221.3 ms from the sensor frame period/acquisition interval through local storage commit, but steady-state execution is pipelined rather than serialized. The latency breakdown involves the sensor frame period/acquisition interval (100 ms, 45.2%), error-bounded compression (68.5 ms, 30.9%), TSN transmission (0.6 ms, 0.3%), and storage operations (~52.2 ms, including encryption; 23.6%).

Although the cumulative latency exceeded the 100 ms sensor period, the evaluated prototype supports steady-state pipelined ingestion within the per-frame budget. This interpretation assumes that compression and storage run on decoupled worker threads, sufficient CPU cores are available, and queues do not accumulate over long horizons; under those conditions, the storage latency of ~51 ms does not block the ingestion of the next sensor frame. This architecture supports flexible storage policies, such as event-triggered logging (e.g., post-incident recording) or periodic backup (e.g., 1 Hz), ensuring that the system’s resources are primarily dedicated to real-time perception tasks while maintaining a secure audit trail. This cumulative value of 221.3 ms should therefore not be interpreted as a serialized per-frame blocking time in steady-state operation. Rather, the steady-state throughput is governed by whether each synchronous stage fits within the 100 ms sensor period. In our measurements, error-bounded compression (68.5 ms), TSN transmission (0.6 ms), and the local storage commit with encryption (~52.2 ms) each complete within this budget, so subsequent frames can be ingested while earlier frames are still traversing downstream stages. Blockchain anchoring is asynchronous and is not part of the real-time control path. In the evaluated PoC, anchoring was executed on a local Hardhat EVM with deterministic instant mining for reproducible gas and negative-test measurements. Public L1/L2 confirmation latency is network-dependent and was not measured in this evaluation.

## 5. Discussion

The experimental results provide feasibility evidence that the framework can reduce bandwidth and storage pressure for ADS-like LiDAR data-management workloads within the evaluated setup. By achieving a 75.4% reduction in LiDAR data volume—which translates to a 53.7% system-wide bandwidth reduction—while maintaining full TSN schedulability in the evaluated network model, controlled information loss appears viable for this data-management setting. Our analysis identifies 2 cm as the practical error bound in which compression efficiency is high and reconstruction quality remains within the selected geometric-fidelity criterion. Under the reported reconstruction metrics, fidelity at this operating point satisfies the selected geometric proxy criteria; this is consistent with the physical scale of objects in driving scenarios, but we do not claim to have directly measured downstream perception performance, which we leave to future work.

Unlike compression-only studies, our work covers the complete data pipeline, including deterministic networking and decentralized storage. The harmonized-period design replaces best-effort networking with the deterministic guarantees that sensor streams require. The hybrid IPFS-blockchain architecture can reduce dependence on a single centralized repository when IPFS objects are properly pinned and replicated, while the current evaluation measures local IPFS ingestion and on-chain anchoring logic rather than large-scale distributed availability. The modular design could allow the compression and TSN components to be adopted incrementally as hardware capabilities evolve.

This end-to-end coverage also yields a design-time co-parameterization benefit: a single declared error bound is a shared control across all three layers. Relaxing the error bound, i.e., allowing a larger ε, reduces the per-frame payload, which directly relaxes the TSN stream set and link utilization presented to the scheduler (Algorithm 1, lines 12–14). Conversely, tightening the error bound increases the payload but corresponds to a stricter declared fidelity tier and a tighter archival policy in the verifiable-archival layer (Section 3.3). This shared error-bound contract—the same parameter shaping the schedulable traffic on one side and constraining the on-chain fidelity tier on the other—is a configuration-level coupling rather than a closed-loop emergent behavior, and closed-loop online adaptation across layers is left to future work. Even at design time, it nonetheless allows the framework to trade bandwidth, latency headroom, and archival fidelity through one consistent control rather than tuning three subsystems independently, which is not possible when the technologies are merely juxtaposed.

Three supplementary analyses further characterize robustness. The cross-scene sensitivity analysis (Section 4.3) shows that scene variation perturbs both the compression ratio and the Occupancy IoU, yet the derived per-stream bitrate and link utilization remain low and stable, so the ε = 2.0 cm operating point remains a practical trade-off across scenes. The companion compression–reconstruction trade-off (Figure 8) confirms that the error bound ε is the dominant application-level control—scene-to-scene variation within each error-bound cluster is noticeably smaller than the effect of changing ε—with the person scene consistently on the favorable end, in keeping with its sparser, more regular geometry. Finally, the background-traffic schedulability check (Section 4.6) confirms that all nine (error-bound, background-load) configurations remain feasible, with nominal link utilization staying well below the 1 Gbps capacity in the evaluated abstraction.

The role of blockchain in this framework deserves to be stated explicitly. In BEDM, blockchain is used for tamper-evident anchoring of content identifiers and for auditability of stored payloads. Because confirmation happens asynchronously and the real-time control path depends only on local processing, TSN delivery, and local IPFS/manifest updates, blockchain is deliberately kept off the safety-critical loop. Lighter-weight alternatives, such as signed centralized logs, would reduce gas cost and improve simple write throughput, but they offer weaker multi-party verifiability and weaker resistance to single-point administrative tampering. The hybrid IPFS-plus-on-chain batch-commitment design chosen here is therefore a trade-off: it accepts transaction cost and confirmation delay in exchange for decentralized, verifiable integrity of high-volume sensor records. It also explains two observations that are otherwise counter-intuitive: LiDAR data volume drops substantially after compression, yet the IPFS add time improves only modestly because the fixed chunking, hashing, and indexing overhead dominates for sub-megabyte payloads; and EB-HC-3D(Axis) and EB-HC-3D(L2) produce nearly overlapping curves because once octree partitioning plus Huffman coding is applied, the remaining entropy of the encoded stream is similar in our data regardless of the distance metric.

The results presented here are specifically supported within the KITTI dataset, the currently measured compression summary, and the single-switch-oriented TSN evaluation. Broader claims for arbitrary autonomous-driving datasets, richer multi-switch or ring topologies, and heavier or more diverse traffic mixes are not directly validated and remain future work.

Several limitations remain. Future work will explore dynamic packet-allocation algorithms that can adapt to variable compression ratios without sacrificing deterministic guarantees. Blockchain transaction costs on mainnets also remain a concern. We plan to investigate Layer-2 solutions such as Rollups and zero-knowledge proofs to reduce costs and strengthen privacy. In addition, a direct evaluation of error-bounded compression on downstream perception tasks—object detection, segmentation, and tracking—is left to future work, since the present analysis relies only on geometric proxies (Occupancy IoU and Chamfer distance); likewise, on-target timing on automotive-grade edge hardware remains future work, as the per-stage latencies reported here were measured on a desktop-class proxy platform. On the networking side, future work should evaluate mixed-periodicity, asynchronous, and sporadic traffic, clock-synchronization error and jitter, bursty cross-traffic, and behavior near link saturation, using packet-level simulation (e.g., OMNeT++/INET) and hardware testbeds with real PTP/IEEE 802.1AS synchronization.

Table 6 expands the Table 5 negative-test results into a threat-vector framing: each row pairs an in-scope attack vector with the BEDM control that detects or prevents it, plus the remaining limitation that orthogonal mechanisms must address. Table 6 is a complementary summary of Table 5 rather than a separate result.

Out-of-scope threats. The following must be addressed by orthogonal mechanisms: LiDAR sensor spoofing and sensor-level false data injection; authorized writers producing semantically false records; full compromise of the AES key or writer private key; denial-of-service on IPFS, the RPC endpoint, or the chain; smart contract bugs beyond the contract presented here; and long-range reorganization or consensus attacks on Ethereum L1. The component improves tamper evidence and auditability of archived records; it does not provide end-to-end sensor security.

## 6. Conclusions and Future Work

We present BEDM, an integrated in-vehicle data-management framework that co-designs error-bounded LiDAR compression, TSN-based deterministic delivery, and blockchain/IPFS-based verifiable archival. Evaluation on the KITTI dataset shows that the integrated pipeline reduces bandwidth and storage demand while preserving TSN schedulability and tamper-evident archival under the stated trust and threat model within the evaluated setup. Although validation is limited to the KITTI dataset and a single-switch setup, the results show internally consistent bandwidth, schedulability, and archival behavior within this evaluated setting. Future work will focus on extending the framework to multi-node vehicular ad hoc network (VANET) architectures, leveraging Layer-2 Rollup technology to improve on-chain transaction throughput, and implementing real-time adaptive compression strategies to dynamically manage varying data traffic. These directions would improve scalability and responsiveness for real-world ADSs.

The asynchronous blockchain/IPFS layer provides verifiable tamper-evident anchoring for encrypted archival records under the trust and threat model stated in Section 3.3. Measured gas costs show that per-frame Ethereum L1 anchoring is financially impractical, while batch anchoring substantially reduces gas demand and can support lower-frequency audit-grade use cases, subject to the deployment chain and fee regime. Layer-2 deployment may further reduce cost, but USD-denominated savings depend on the selected network and market conditions. The component does not address sensor-level threats and is not part of the real-time control path; it is presented as one integrated layer of an end-to-end pipeline whose primary contribution is the cross-layer combination of error-bounded compression, deterministic TSN delivery, and verifiable archival.

Beyond in-vehicle ADSs, the framework points toward a broader pattern in IoT data engineering: jointly optimizing data processing, deterministic transmission, and privacy-preserving storage for high-volume sensor streams.

## Figures and Tables

**Table 1:** Comparison of representative studies with BEDM. Note: ✓ indicates that the feature is supported; – indicates that the feature is not addressed.

Previous Work	Main Idea	Bounded-Error Compression	LiDAR-Specific	TSN Layer	Blockchain Layer
SZ/ZFP [23,24]	Foundational bounded-error compression for scientific floating-point data	✓	–	–	–
Chang et al. [7]	Bounded-error LiDAR compression with user-defined coordinate deviation	✓	✓	–	–
Craciunas et al. [11]	Offline schedule synthesis for IEEE 802.1Qbv networks	–	–	✓	–
Ye and Park [12]	Vehicle-data storage and sharing via blockchain and IPFS	–	–	–	✓
Chen et al. (TECChain) [29]	Blockchain-based collaboration management in TSN	–	–	✓	✓
This work (BEDM)	Integrated pipeline for bounded-error LiDAR compression, TSN-based delivery, and blockchain/IPFS archival	✓	✓	✓	✓

**Table 2:** Comparison of compression strategies.

Method	Summary	Time Complexity
Huffman	Frequency-based byte encoding (baseline)	O(N)
EB-HC (Axis)	Merge integer coords along x, y, z + Huffman	O(NlogN)
EB-HC (L2)	Merge integer coords in 3D distance + Huffman	O(NlogN)
EB-3D (Axis)	Octree subdivision (per dimension)	O(N×depth)
EB-3D (L2)	Octree subdivision (L2 distance check)	O(N×depth)
EB-HC-3D (Axis)	Combine octree + Huffman (Axis-based)	O(N×depth)
EB-HC-3D (L2)	Combine octree + Huffman (L2-based)	O(N×depth)

**Table 3:** Cross-scene sensitivity of EB-HC-3D(L2) under different error-bound settings. Values are unweighted across-scene means over the five KITTI scene groups (campus, city, person, residential, road). Values after ± are 95% confidence intervals across the five scene means (Student’s t, *n* = 5 scenes).

ε (cm)	Mean Compression Ratio	Mean Occupancy IoU	Derived Bitrate (Mbps)	Link Utilization (%)
0.5	0.2893 ± 0.0200	0.9452 ± 0.0030	45.72	4.57
1.0	0.2595 ± 0.0208	0.8953 ± 0.0079	40.95	4.10
2.0	0.2309 ± 0.0205	0.8051 ± 0.0114	36.44	3.64

**Table 4:** TSN scheduler comparison on the Section 4.5 six-stream flow set (single- and two-switch topologies). Both schedulers operate at the same 20.49% link utilization (identical 2 cm operating-point traffic) and meet all per-flow deadlines; the reported metrics follow the benchmarking methodology of [30].

Scheduler	Schedulable (Single-Switch)	Solve Time (Single-Switch)	Schedulable (Two-Switch)	Solve Time (Two-Switch)
No-wait list scheduling [10]	✓	~0.04 s	✓	~0.04 s
SMT-based exact [11]	✓	~0.06 s	✓	~0.08 s

**Table 5:** Executable negative tests; all nine passed.

Test	Attack	Defense
NT-1	Tampered ciphertext	AES-GCM tag
NT-2	Tampered metadata (AAD)	AES-GCM tag
NT-3	Non-writer anchorBatch	onlyWriter modifier
NT-4	Replay (duplicate batchId)	anchoredAt-zero check
NT-5	Forged Merkle leaf	verifyRecord returns false
NT-6	Manifest CID substitution	on-chain manifestCidHash
NT-7	Error bound above tier bound	errorBoundDmm ≤ tier bound
NT-8	Writer-tier escalation	tierId ≥ writerMinTier
NT-9	Window above tier max	window ≤ maxBatchWindowSec

**Table 6:** Threat-specific security analysis: in-scope attack vector, BEDM mitigation, and remaining limitation requiring orthogonal mechanisms.

Attack Vector	Mitigation in BEDM	Remaining Limitation
Altered IPFS ciphertext/metadata	CID + object hash + AES-GCM tag (AAD-bound)	IPFS availability requires pinning
Unauthorized record submission	onlyWriter + writer key	key compromise needs rotation
Record replay/reordering	frame_id, previous_record_id, Merkle binding	depends on writer honesty
Manifest CID substitution/forged batch	on-chain manifestCidHash + verifyRecord	finality/reorg needs a confirmation policy
Local log deletion after incident	IPFS pinning + chain anchor	lost replicas not reconstructable
Tier escalation by writer	tierId ≥ writerMinTier + errorBoundDmm ≤ tier bound	contract does not verify actual reconstruction error
Anchoring-policy abuse (spam/window inflation)	minAnchorIntervalSec + maxBatchWindowSec per tier	does not bound off-chain retention

## Data Availability

The data are contained within the article. The Solidity source code of the BEDMAnchor smart contract and its executable negative-test suite are available from the corresponding author upon reasonable request.

## Abbreviations

The following abbreviations are used in this manuscript:

ADS	Autonomous Driving System
AES	Advanced Encryption Standard
AES-GCM	Advanced Encryption Standard—Galois/Counter Mode
BEDM	Blockchain-based Error-bounded Data Management
CID	Content Identifier
DAG	Directed Acyclic Graph
EB	Error-Bounded
EB-3D	Error-Bounded 3D Compression
EB-HC	Error-Bounded Huffman Coding
EB-HC-3D	Error-Bounded Huffman Coding with 3D Integration
ECU	Electronic Control Unit
GCL	Gate Control List
G-PCC	Geometry-based Point Cloud Compression
HPC	High-Performance Computing
IEEE	Institute of Electrical and Electronics Engineers
IoT	Internet of Things
IPFS	InterPlanetary File System
KITTI	Karlsruhe Institute of Technology and Toyota Technological Institute
LiDAR	Light Detection and Ranging
MPEG	Moving Picture Experts Group
P2P	Peer-to-Peer
PCL	Point Cloud Library
PTP	Precision Time Protocol
SHA	Secure Hash Algorithm
SMT	Satisfiability Modulo Theories
TAS	Time-Aware Shaper
TSN	Time-Sensitive Networking
VANET	Vehicular Ad hoc Network
